# GiniClust: detecting rare cell types from single-cell gene expression data with Gini index

**DOI:** 10.1186/s13059-016-1010-4

**Published:** 2016-07-01

**Authors:** Lan Jiang, Huidong Chen, Luca Pinello, Guo-Cheng Yuan

**Affiliations:** Department of Biostatistics and Computational Biology, Dana-Farber Cancer Institute, Boston, MA 02215 USA; Department of Biostatistics, Harvard T.H. Chan School of Public Health, Boston, MA 02115 USA; Boston Children’s Hospital, Boston, MA 02115 USA; Department of Computer Science and Technology, Tongji University, Shanghai, China; Harvard Stem Cell Institute, Cambridge, MA 02138 USA

**Keywords:** Clustering, Single-cell analysis, RNA-seq, qPCR, Gini index, Rare cell type

## Abstract

High-throughput single-cell technologies have great potential to discover new cell types; however, it remains challenging to detect rare cell types that are distinct from a large population. We present a novel computational method, called GiniClust, to overcome this challenge. Validation against a benchmark dataset indicates that GiniClust achieves high sensitivity and specificity. Application of GiniClust to public single-cell RNA-seq datasets uncovers previously unrecognized rare cell types, including Zscan4-expressing cells within mouse embryonic stem cells and hemoglobin-expressing cells in the mouse cortex and hippocampus. GiniClust also correctly detects a small number of normal cells that are mixed in a cancer cell population.

**Electronic supplementary material:**

The online version of this article (doi:10.1186/s13059-016-1010-4) contains supplementary material, which is available to authorized users.

## Background

Multicellular organisms are composed of diverse cell types with distinct morphologies and functions. Characterizing their differences is essential for both basic developmental biology research and clinical diagnosis and treatment of human diseases. There has yet to be a uniformly accepted standard for cell-type classification, but it has become increasingly appreciated that analysis of global gene expression patterns provides a systematic and functional basis [1]. However, traditional microarray and RNA-seq technologies can only profile the average gene expression level among a large cell population that often contains significant heterogeneity. As a result, it is likely that many cell types remain unrecognized.

The recently developed single-cell genomics and proteomics technologies have provided a new opportunity. Specialized computational methods have been developed to identify cell types from single-cell gene expression data [2, 3]. Applications of these technologies have led to the discovery of many unrecognized cell types in diverse tissues, including the hematopoietic, neural, immune, and digestion systems, and as also to improved characterization of cancer heterogeneity [4–10].

Cell types that play an important role in development or disease progression often have low abundance. Examples of such rare cell types include stem and progenitor cells [11], cancer stem cells [12], and circulating tumor cells [13]. To date, systematic identification of rare cell types from single-cell gene expression data remains a major challenge. Among the aforementioned methods, only RaceID [8] is designed specifically to identify rare cell types.

In this paper, we develop a new algorithm, called GiniClust, for rare cell type detection and show that it outperforms RaceID for both simulated and biological datasets. The most important feature in GiniClust is a novel gene selection method that is particularly suitable for rare cell type identification, borrowing ideas from the social science domain. We apply GiniClust to a number of public datasets and gain new biological insights.

## Results

### Overview of the GiniClust method

**Fig. 1:**
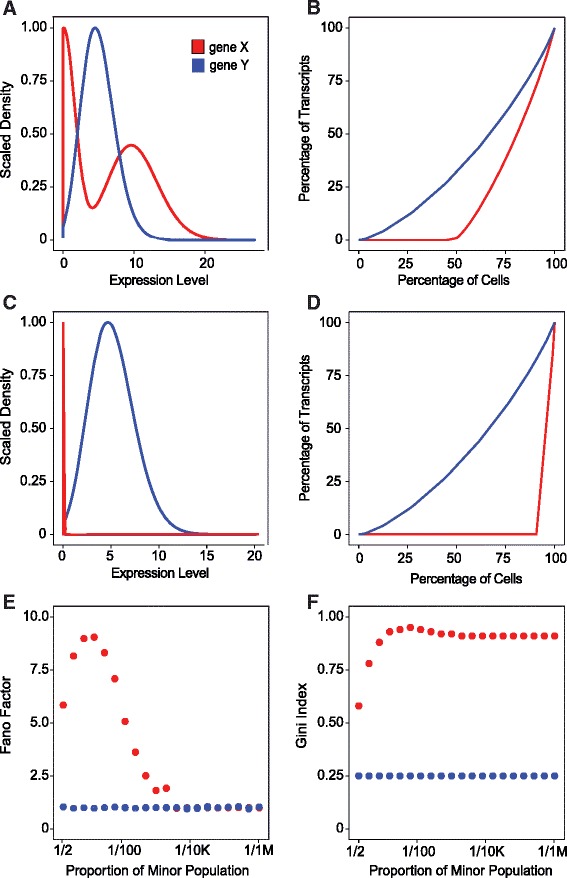
Coparison between Gini index and Fano factor in detecting differentially expressed genes. **a** Scaled density plot of the expression levels of genes X (*red*) and Y (*blue*). The proportion of the minor cell type is 50 %. **b** The Lorenz curve for genes X (*red*) and Y (*blue*). The proportion of the minor cell type is 50 %. **c, d** Same as (**a, b**), except the proportion of the minor cell type is changed to 1e-5. **e** Fano factor for genes X and Y for varying proportions of the minor cell type (1/1 M stands for one in one million). **f** Gini index for genes X and Y for varying proportions of the minor cell type

To motivate GiniClust, we first note that cell clustering is dependent on the selection of genes. Traditionally one often uses the most variable genes for clustering [14]. For single-cell RNA-seq data, a commonly used metric for variability is the Fano factor, defined as the ratio between the variance and the mean [15]. To illustrate its limitation in identifying rare cell types, we consider the following hypothetical example. Consider a mixed population of 1,000,000 cells containing two cell types. We examine the expression patterns associated with two genes, X and Y, where only X is differentially expressed. If 50 % of the population is obtained from either cell type, then the Fano factor of X is much higher than that of Y (Fig. 1a). This property of differentially expressed genes is the main premise underlying variance-based gene selection methods. However, as the cell population becomes increasingly imbalanced, the difference between X and Y becomes much smaller (Fig. 1c, e). When the fraction of the minor cell type is less than 0.01 %, there is essentially no difference between the Fano factor values for X and Y, indicating that the Fano factor is not suitable for selecting rare cell-type-specific genes.

To overcome this limitation, we have developed a new approach to systematically identify genes that are specific to rare cell types. The Gini index [16], which was originally developed to study social inequality, has been used to identify countries whose wealth is concentrated by a small number of individuals (http://data.worldbank.org/indicator/SI.POV.GINI/) and is particularly suitable for identifying rare cell-type-specific genes. For each gene X, we sort cells based on its expression levels from the lowest to the highest and then evaluate the cumulated expression levels of X as more and more cells are included from the ranked list. A plot of this functional relationship is called the Lorenz curve (Fig. 1b, d). The Gini index is defined as two times the area between the Lorenz curve and the diagonal. The value of the Gini index varies between 0 (most uniform) and 1 (most extreme).

To demonstrate the utility of the Gini index, we reexamine the above simulated example. As shown in Fig. 1f, the difference between the Gini index values associated with X and Y increases substantially as the minor cell type becomes less abundant, and the difference persists over a wide range of mixing frequencies.

**Fig. 2:**
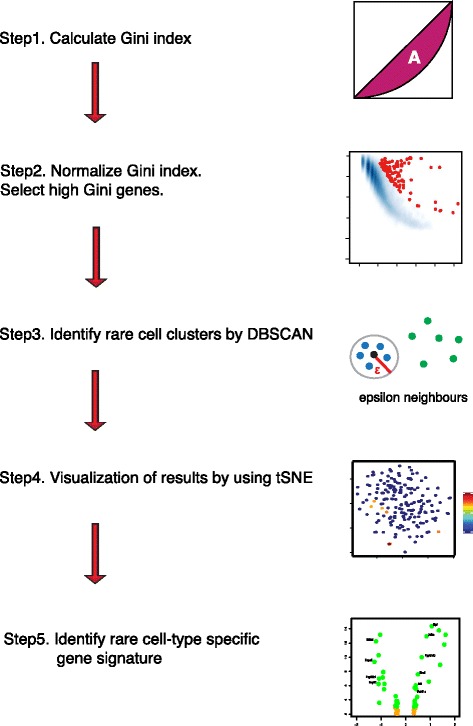
Overview of the GiniClust pipeline. Details are described in Methods

Figure 2 shows a schematic of the pipeline of our method, named GiniClust, for detecting rare cell types. We have made several modifications of the Gini index to enhance its utility for detecting rare cell-type-specific genes. First, we define a bidirectional Gini index to identify genes that are specifically unexpressed in a rare cell type (this extension is used only for qPCR data analysis but not for RNA-seq data analysis, as explained later). Second, we normalize the Gini index values to remove a systematic bias toward lowly expressed genes. After normalization, the genes with highest Gini index values are selected for further analysis and referred to as high Gini genes. Based on the expression profile of the high Gini genes, we identify cell clusters by using the algorithm density-based spatial clustering of applications with noise DBSCAN [17]. Two additional steps are added to interpret the clustering results. First, we use t-distributed stochastic neighbor embedding (t-SNE) [18], a nonlinear dimensionality reduction method, to examine whether identified clusters are visually distinct. Second, we use differential gene expression analysis to identify the gene signature associated with each detected rare cell type. The details of the GiniClust pipeline are described in the Methods section.

### GiniClust accurately identifies cell subpopulations from qPCR data

**Fig. 3:**
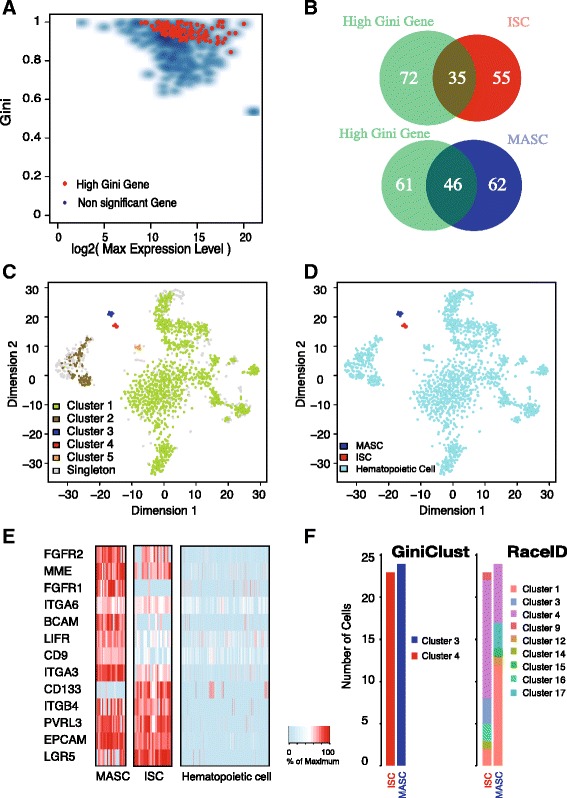
GiniClust uncovers rare cell types from the qPCR dataset. **a** Relationship between the raw Gini index and the log2-transformed maximum expression level. Selected genes with high normalized Gini index values are labeled as *red dots*. **b** Overlap between the selected high Gini genes and differentially expressed genes. **c** t-SNE visualization of the data. Cells are color-coded based on the GiniClust cluster membership. **d** t-SNE visualization of the same data as in **c**. Cells are color-coded based on the actual lineage. **e** Expression levels of representative genes for MASC (*n* = 24), ISC (*n* = 23), and other cells (*n* = 1916). Gene expression levels are normalized as percentage of the corresponding maximum values. **f** Comparison between GiniClust and RaceID in detection of ISC and MASCs in the mixture of cells

We started by testing whether GiniClust can accurately detect rare cell types of a known origin. To this end, we analyzed a multiplex qPCR dataset generated from a previous study [9]. The dataset consists of the expression levels of 280 common cell surface markers in 1916 cells extracted from the mouse hematopoietic system, as well as 24 mammary gland stem cells (MASCs) and 23 intestinal stem cells (ISCs). All cells were profiled by using the same set of primers; therefore, their gene expression patterns were directly comparable. We computed the bidirectional Gini index values in order to identify genes that were either upregulated (direction = 1) or downregulated (direction = –1) in rare cell populations (see Methods for details). After normalization, we identified 107 high Gini genes (normalized Gini index value >0.05) (Fig. 3a, Additional file 1: Table S1), including well-known MASC markers such as *MME* (*CD10*), *FGFR1*, and *FGFR2*, and ISC markers such as *LGR5*, *EPCAM*, and *CD133*. Among this list, 46 genes (42.59 %) were differentially expressed (fold change >2 and *p* value < 1e-5, two-sample *t* test) between ISC and hematopoietic cells, and 35 genes (38.9 %) were differentially expressed between MASC and hematopoietic cells (Fig. 3b, e, Additional file 2: Table S2), although the enrichment was not statistically significant.

By using a correlation-based distance to compare cell similarity, GiniClust identified five clusters (Fig. 3c, Additional file 3: Table S3) containing two major clusters and three rare clusters. To be precise, here we define rare cell clusters as those that consist of less than 5 % of the total cell population. In addition, 311 cells were annotated as singletons that were isolated from all other cells therefore could not be reliably assigned to any cluster. We label each cluster simply based on its relative size; therefore, Cluster 1 is the largest cluster, while rare clusters are listed in the end. The two major clusters, Cluster 1 (1452 cells) and Cluster 2 (144 cells), are all composed of hematopoietic cells, with Cluster 2 associated with elevated *CD3* and *CD25* expression. Cluster 3 and Cluster 4 exactly match the MASCs and ISCs (Fig. 3d and f), respectively. Cluster 5 contains 8 cells and is characterized by elevated *IL7R* expression. To functionally characterize the cell type associated with Cluster 5, we compared its gene expression pattern with that of Cluster 1 and identified 20 genes specifically expressed in Cluster 5 (fold change >1.5). We then applied functional enrichment analysis using DAVID (https://david.ncifcrf.gov/) and found that this gene list was highly enriched for “immune systems process” (*p* value = 3.0e-11) and “cell-cell adhesion” (*p* value = 1.5e-10), suggesting that the cells in Cluster 5 may be involved in immune responses. t-SNE plots show that these clusters are well separated from each other (Fig. 3c, d).

For comparison, we analyzed the same dataset by using RaceID [8], a recently developed computational method for rare cell type detection. RaceID identified 22 clusters, including 19 rare cell clusters. Unlike GiniClust, with RaceID both MASCs and ISCs contain cells from multiple clusters. In addition, each ISC- (or MASC)-containing cluster consists of cells with multiple cell lineages (Fig. 3f and Additional file 4: Figure S1). These observations indicate that RaceID is less accurate than GiniClust. We further compared the performance of RaceID and GiniClust using a simulated single-cell RNA-seq dataset, which contained two major clusters and three rare cell clusters. Each major cluster contained 1000 cells, whereas the rare cell clusters contained 4, 6, and 10 cells, respectively (see Methods for details). Again, GiniClust identified the three rare cell clusters perfectly. On the other hand, RaceID correctly detected the rare cells as outliers but assigned them to incorrect clusters (Additional file 5: Figure S2).

Taken together, the preceding results strongly indicate that GiniClust is effective for detecting rare cell types and outperforms existing methods. Therefore, we are interested in applying GiniClust to discover novel cell types from a number of recently published datasets, as discussed in the following sections.

### GiniClust identifies Zscan4-enriched rare cluster from mouse embryonic stem cells

**Fig. 4:**
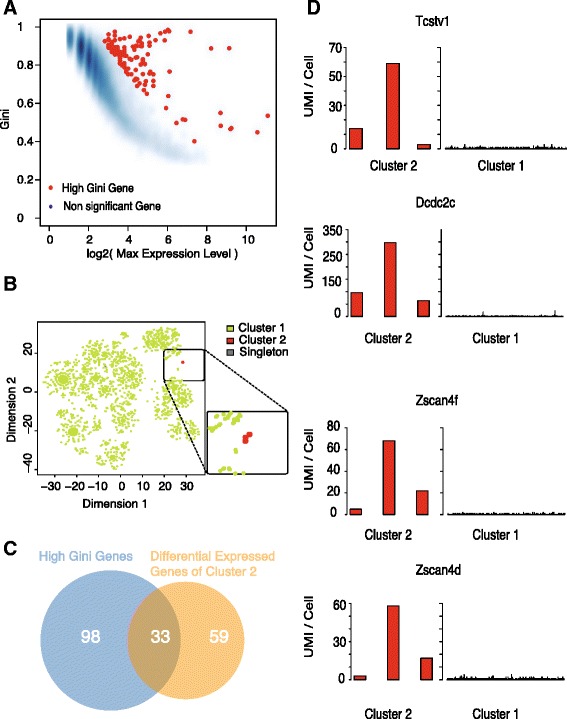
GiniClust identifies a Zscan4-enriched rare cluster from mouse embryonic stem cells. **a** Relationship between the raw Gini index and the log2-transformed maximum expression level. Selected genes with high normalized Gini index values are labeled as *red dots*. **b** t-SNE visualization of the data. Cells are color-coded based on the GiniClust cluster membership. Inset shows a zoomed-in region around the rare cell cluster. **c** Overlap between the selected high Gini genes and upregulated genes in Cluster 2. **d** Expression pattern of representative genes (*Tcstv1*, *Dcdc2c*, *Zscan4f*, *Zscan4d*) in Cluster 2 (*n* = 3, *left* panels) compared to Cluster 1 (*n* = 2505, *right* panels). Each bar represents a single cell

In the first dataset we analyzed, mouse embryonic stem cells (ESCs) were assayed by using a droplet-based high-throughput sequencing technology called inDrop at three time points: Day 0, Day 2, and Day 4 after leukemia inhibitory factor (LIF) removal induced differentiation [19]. We focused on a subset of 2509 cells obtained from the Day 0 stage, where the cells remained undifferentiated. On average, about 13,000 unique molecular identifiers (UMIs) were detected in each cell, corresponding to nearly 6000 genes. Since single-cell RNA-seq technologies have low detection efficiency, it is possible that a gene can be undetected in a cell simply due to technical artifacts such as dropout [20]. Since we cannot reliably detect genes that are specifically downregulated in a rare cell type, we evaluated one-direction Gini index values to select high Gini genes using a standardized pipeline for parameter selection (see Methods for details). A total of 131 high Gini genes (Fig. 4a, Additional file 6: Table S4) were selected (*p* value < 0.0001). Using the Jaccard distance as the metric for comparing cell similarity, GiniClust identified two clusters (Fig. 4b, Additional file 7: Table S5). Nearly all (99.8 %) cells were assigned to Cluster 1, whereas Cluster 2 contained only 3 cells. Only one cell was annotated as a singleton. The t-SNE plot confirms that the two clusters were well separated (Fig. 4b).

The number of cells in Cluster 2 is very small. To exclude the possibility of technical artifacts, we tested whether this result could be reproduced for resampled data. To this end, we randomly sampled from non-rare cells while keeping Cluster 2 intact and then applied GiniClust to identify rare clusters. We repeated this analysis five times, varying the sampling frequency from 50–90 % in 10 % intervals. In each case, GiniClust precisely re-identified Cluster 2 as a rare cell cluster, suggesting that the result is highly robust.

By comparing the gene expression patterns between the two clusters, we identified 77 differentially expressed genes (MAST [21] likelihood ratio test *p* value < 1e-5; fold change >2) (Additional file 8: Table S6). Among these differentially expressed genes, 33 were high Gini genes (Fig. 4c). This overlap between the two gene lists is statistically significant (Fisher exact test, *p* value < 2.2e-16). Strikingly, several genes were expressed at an extremely high level in Cluster 2, but had very low expression in Cluster 1, including a number of genes from the *Zscan4* gene family (Fig. 4d, Additional file 8: Table S6). Expression of these genes has previously been observed in the two-cell embryo stage and 2C-like cells [22], although their potential function in pluripotency remains unknown.

In order to test whether Cluster 2 shares similar transcriptomic profiles to those in 2C-like cells, we extracted a 2C-like gene signature from the literature [22] and quantified its similarity with each cell in the inDrop dataset by defining a 2C-like score (see Methods for details). Strikingly, the 3 cells from Cluster 2 were ranked at the top and distinct from all other cells (Additional file 9: Figure S3). This analysis suggests that Cluster 2 may indeed have totipotent properties like those of 2C-like cells.

### GiniClust identifies rare normal cells in glioblastoma samples

**Fig. 5:**
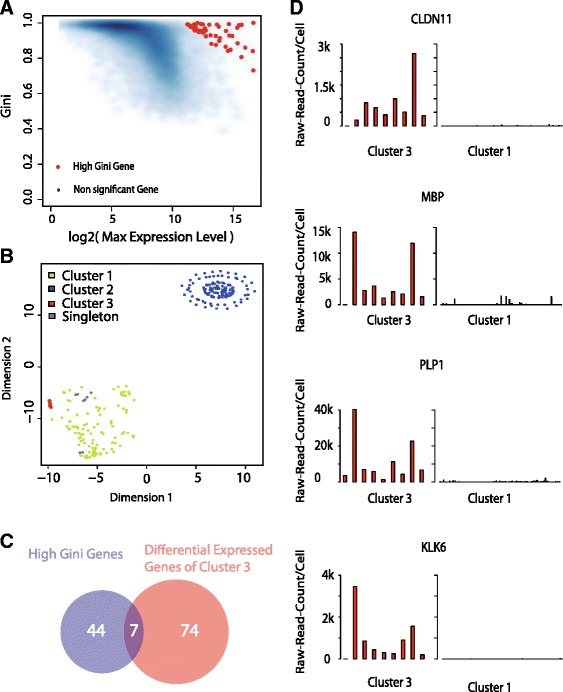
GiniClust identifies a rare cluster in glioblastoma samples. **a** Relationship between the raw Gini index and the log2-transformed maximum expression level. Selected genes with high normalized Gini index values are labeled as *red dots*. **b** t-SNE visualization of the data. Cells are color-coded based on the GiniClust cluster membership. **c** Overlap between the selected high Gini genes and upregulated genes in Cluster 2. **d** Expression pattern of representative genes (*CLDN11*, *MBP*, *PLP1*, *KLK6*) in Cluster 3 (*n* = 9, *left* panels) compared to Cluster 1 (*n* = 261, *right* panels). Each bar represents a single cell

Next, we analyzed a single-cell RNA-seq dataset obtained from glioblastoma (GBM) primary tumors (576 cells) [23]. We identified 51 high Gini genes (Fig. 5a, Additional file 10: Table S7). Based on this gene list, GiniClust identified 3 clusters (Fig. 5b, Additional file 11: Table S8). Ten cells were labeled as singletons. The rare cluster, Cluster 3, contains 9 cells originating from two different tumors, MGH31 and MGH29. We found 81 genes that were significantly upregulated (MAST likelihood ratio test *p* value < 1e-5, fold change >2) in Cluster 3 compared to Cluster 1 (Additional file 12: Table S9). Again, the overlap between the two gene lists is statistically significant (Fisher exact test, *p* value 1.3e-9) (Fig. 5c).

Several genes that were highly expressed in Cluster 3 were well known to be preferentially expressed in normal oligodendrocytes, including *CLDN11*, *MBP*, *PLP1*, and *KLK6* (Fig. 5d), indicating that these cells are not cancer cells. Such cells were also detected in the original study but only through using extensive biological knowledge [23]. Such knowledge is not required in GiniClust analysis.

### GiniClust identifies an undetected rare cell type in mouse somatosensory cortex

Finally, we analyzed a third single-cell RNA-seq dataset containing 3005 single cells obtained from the mouse somatosensory cortex and hippocampus CA1 region [4]. The authors developed a computational tool called BackSPIN and applied it to identify 47 clusters. The number of cells in each cluster varied from 5 to 380.

**Fig. 6:**
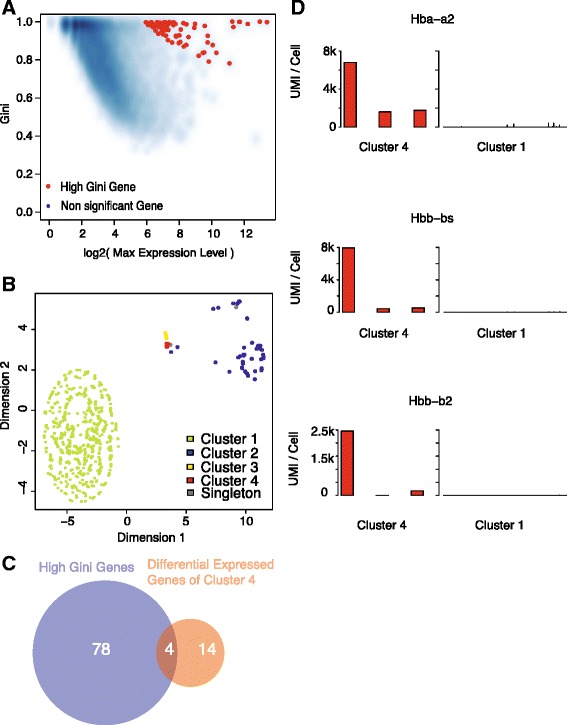
GiniClust identifies a rare cell type in mouse cortex and hippocampus. **a** Relationship between the raw Gini index and the log2-transformed maximum expression level. Selected genes with high normalized Gini index values are labeled as *red dots*. **b** t-SNE visualization of the data. Cells are color-coded based on the GiniClust cluster membership. **c** Overlap between the selected high Gini genes and upregulated genes in Cluster 4. **d** Expression pattern of representative genes (*Hba-a2*, *Hbb-b2*, *Hbb-bs*) in Cluster 4 (*n* = 3, *left* panels) compared to Cluster 1 (*n* = 1842, *right* panels). Each bar represents a single cell. The expression levels of *Hba-a2* shown here represent the sum of the levels of *Hba-a2_loc1* and *Hba-a2_loc2* in the original paper

We identified 82 high Gini genes (Fig. 6a, Additional file 13: Table S10). Based on this gene list, GiniClust identified 4 cell clusters (Fig. 6b, Additional file 14: Table S11), including two rare clusters (Cluster 3 and Cluster 4). Six cells were annotated as singletons. Cluster 3 contains 76 cells, 74 (97.4 %) of which were annotated as interneurons by BackSPIN. Compared to Cluster 1, Cluster 3 highly expresses *Gad2* and *Gad1*. On the other hand, Cluster 4, which contains only 3 cells, overlaps with three distinct clusters identified by BackSPIN (Additional file 15: Figure S4). To gain functional insights, we compared its transcriptomic profile with Cluster 1 and identified 18 upregulated genes (fold change >2; MAST likelihood ratio test *p* value < 1e-5)(Additional file 16: Table S12). The overlap with high Gini genes is statistically significant (Fisher exact test, *p* value = 2.2e-6, Fig. 6c). Of note, there are 3 hemoglobin genes, including *Hba-a2*, *Hbb-bs*, and *Hbb-b2*, that are highly expressed in Cluster 4 but not expressed elsewhere (Fig. 6d), raising an interesting question of whether hemoglobin genes play a functional role in a subset of neurons.

## Discussion and conclusions

There is a large body of literature in clustering analysis [24]. Traditional clustering methods are effective for identifying large clusters but are not suitable for detecting rare cell clusters, mainly because the feature selection is insensitive to the presence of rare cell clusters. We have proposed to use the Gini index as the basis to select rare cell-type-specific genes and have shown that this approach is effective in all the datasets analyzed here.

Our analysis of single-cell RNA-seq datasets has identified rare cell types that were not previously recognized. First, in mouse embryonic stem cells, we found a cluster of 3 cells that highly expressed *Zscan4* genes, indicating that mouse ESCs contain a rare subpopulation that has greater differentiation potential than commonly thought. Of note, the expression of these genes was previously observed on Day 2 and Day 4 after LIF removal induced differentiation, but not in undifferentiated cells. Second, in the mouse cortex and hippocampus, we identified a cluster (Cluster 4) of 3 cells that highly express several hemoglobin genes, including *Hba-a2*, *Hbb-bs*, and *Hbb-b2*. This cluster was not detected in the original study. Expression of hemoglobin genes is commonly thought to be a unique property of erythroid cells, but recent studies have found that they can also be expressed in dopaminergic neurons [25]. It will be interesting to investigate the biological function of hemoglobin expression cells in the mouse cortex in future studies. Although these clusters contain a very small number of cells, they can be robustly detected from resampled datasets (Methods). Of note, it is possible that gene expression pattern differences between clusters may be attributed to mechanisms other than cell-type differences, such as the variation of niche. As such, further experimental investigations are required to functionally test computational predictions.

GiniClust differs from RaceID in a number of significant ways. First, the gene selection method is different. Whereas RaceID selects genes based on the variance of expression levels, GiniClust uses the Gini index to select genes. We have shown that the Gini index is more effective for selecting cell-type-specific genes. Second, RaceID identifies rare cell types using a two-step procedure. First, the cell population is divided into a number of large clusters by using *k*-means. Next, outliers are detected within each cluster. In contrast, in GiniClust all clusters are identified in a single step. Third, RaceID allows a single outlier cell to be identified as a rare cell type, which may explain why it tends to over-cluster. On the other hand, GiniClust requires that each cluster must contain multiple cells. Our analysis of simulated and real datasets suggests that GiniClust is more robust and accurate than RaceID. GiniClust is also much faster than RaceID. For the simulated dataset analyzed in this paper, it took 42 seconds to finish the GiniClust analysis, compared to 7.3 hours using RaceID on the same computer.

A major limitation of GiniClust is that it is not effective for large cluster detection. For example, in our analysis of the simulated data, GiniClust merged the two major clusters into a single big cluster. This occurs because differentially expressed genes between major cell clusters are typically not high Gini genes. One simple yet suboptimal solution is to combine GiniClust with another traditional clustering method, identifying rare clusters first by using GiniClust and then applying the other method to identify large clusters. In future work, we will extend GiniClust to systematically address this limitation.

In summary, we have shown that GiniClust is a powerful tool for detecting rare cell types in normal tissues and disease samples and will facilitate the analysis of single-cell data.

## Methods

### Details of the hypothetical example

We considered a population of 1,000,000 cells consisting of two cell types: major and minor, and two genes: X, a differentially expressed gene, and Y, an undifferentially expressed gene. Within each cell type, the expression levels followed a Poisson distribution determined by a single parameter λ. We set λ_X_ = 0.1 (and 10, respectively) for the major (and minor, respectively) cell type, and λ_Y_ = 5 for both cell types. We varied the proportion of the minor cell type in the population from 0 to 0.5, and calculated the corresponding Fano factor and Gini index for each gene.

### Data sources, preprocessing, and normalization

The mouse qPCR data were obtained from our previous study [9] and processed as described previously [10]. Briefly, gene expression levels were estimated by subtracting the Ct values from the background level of 28, which approximates log2 gene expression levels. Ct values higher than 28 are converted to zero (no expression).

The processed mouse ESC inDrop data were obtained from GSE65525 [19]. The expression level of each gene was represented by UMI-Count/Cell. Genes that were expressed in less than three cells were excluded, leaving 22,830 genes for further analysis. Cells expressing less than 2000 genes were excluded. A total of 2485 cells passed this filter.

The GBM single-cell RNA-seq data were obtained from GSE57872 [23]. Raw sequence reads were mapped to the hg19 reference genome by STAR [26] (version STAR_2.4.2a, option genomeSAindexNbases 14, genomeChrBinNbits 18, genomeSAsparseD 1, sjdbOverhang 75) and quantified by using htseq-count [27] (option --format = bam --order = pos --type = exon --idattr = gene_name). The expression level of each gene was quantified by Raw-Read-Count/Cell. We then applied the same filtering procedure as above to select cells and expressed genes, resulting in a data matrix containing 17,970 genes and 477 cells in total.

The processed mouse cortex single-cell RNA-seq data were obtained from GSE60361 [4]. The gene expression levels were quantified by UMI-Count/Cell. After applying the aforementioned filtering process, we obtained a data matrix containing 15,153 genes and 2545 cells.

### Details of GiniClust pipeline

The GiniClust pipeline contains five steps after data preprocessing (Fig. 2).

1. *Calculate Gini index.*
  The Gini index is calculated based on the normalized gene expression levels. As described in the main text, the Gini index is defined as two times the area between the Lorenz curve and the diagonal. For the qPCR data, we find it useful to define a bidirectional Gini index, which is the maximum value of the positive and negative Gini indexes, as defined below. First, the expression levels are exponentially transformed; that is, a value of *x* is transformed to 2^*x*^ so that it is approximately linearly proportional to the transcript level. The positive Gini index is calculated based on the transformed data. The negative Gini index is defined in a similar manner but using a different transformation: *x* to 2^−*x*^. The bidirectional Gini index is useful for identifying genes that are either upregulated (direction = 1) or downregulated (direction = –1) in rare cells. For RNA-seq analysis, only the positive direction is used for calculating Gini index values since most genes are detected at low levels.
2. *Normalize Gini index. Select high Gini genes*.
  We noticed that the Gini index values are strongly correlated with max gene expression levels (Figs. 1a, 3a, 4a, 5a, 6a); therefore, we devised a normalizing procedure to remove this trend. We used a two-step curve fitting strategy [28] in order to enhance robustness against outliers. Specifically, we first fit a smooth curve through all data points by LOESS regression, removed outliers (defined as those data points for which the residues are above the 75th percentile), and then used LOESS to refit another smooth curve through the remaining data points. LOESS regression was implemented by using the loess function in R. For each gene, we calculated its normalized Gini index value by subtracting the original value by the fitted trend. For RNA-seq data, we further estimated *p* values based on a normal distribution approximation and used the cutoff value (*p* = 0.0001) to select high Gini genes. For qPCR data analysis, we cannot reliably estimate *p* values due to the insufficient number of genes; therefore, we select high Gini genes by thresholding the normalized Gini index values (cutoff value = 0.05).
3. *Identify rare cell clusters by DBSCAN*
  A number of distance metrics may be used for clustering, depending on the statistical property of the gene expression data. For qPCR data, we find that the one-minus correlation metric is suitable because the expression levels can be accurately measured over a wide dynamic range. For RNA-seq data, we find that the Jaccard distance typically generates more robust clustering results.
  We use DBSCAN [17] to cluster cells, as implemented by the dbscan function in the R package fpc, with the method = “dist” setting. For the qPCR dataset, we set eps = 0.25 and MinPts = 5. We use a standardized parameter setting to analyze all real and simulated RNA-seq datasets with MinPts = 3, eps = 0.5. To test robustness, we varied the parameters over a range of values and found that the results are not significantly affected.
  One unique feature of the DBSCAN is that some of the cells, which we call singletons, are not assigned to any cell cluster. The number of singletons detected decreases as the value of eps increases. While both singletons and rare cell types are outliers, the important difference is that a rare cell type contains multiple cells that share similar gene expression patterns.
4. *Visualize results by using t-SNE*
  We use t-SNE for data visualization purposes only, as implemented in the *Rtsne* package in R. The high-dimensional gene expression data are projected into a 2D space, with the “pca = FALSE, max_iter = 3000, and perplexity = 10” setting. The data points are color-coded by using the clustering membership.
5. *Identify rare cell-type-specific gene signature.*
  Rare cell-type-specific gene signatures are identified by using differential expression analysis. For qPCR data, differentially expressed genes are identified by using the two-sided *t* test; for RNA-seq data, differentially expressed genes are identified by using the zlm.SingleCellAssay function in the R package MAST [21], with setting Method = “glm”. The *p* values are calculated by using the hurdle model in the lrTest function in the MAST package.

### Analysis of 2C-like gene signature

We obtained a list of genes that are differentially expressed between 2C and ESCs from [22] and filtered out those genes that were not detected by inDrop. The remaining list contains 65 genes. We define a 2C-like gene signature based on the gene expression pattern of these remaining 65 genes, where each gene is associated with a weight equal to the fold change value between 2C and ESC (extracted from Additional file 7: Table S5 in [22]).

In order to compare the 2C-like gene signature with the single-cell gene expression data, we defined a 2C-like score for each cell in the inDrop dataset as follows. First, the UMI counts were log2-transformed and converted to *z*-scores. Next, we evaluated the inner product between the transformed *z*-score values corresponding to the 65 differentially expressed genes and the aforementioned 2C-like gene signature. Since the average *z*-score value is equal to zero, the mean value of the 2C-like score is also zero for typical ESCs. On the other hand, a 2C-like cell is associated with high 2C-like scores.

### Analysis with RaceID

RaceID was applied to analyze the qPCR dataset as well as simulated data. R scripts of RaceID were downloaded from https://github.com/dgrun/RaceID. We set the model parameters at default values, whereas the number of clusters was set to be 30.

For the qPCR dataset, the Ct-based gene expression levels were exponentially transformed so that they were approximately proportional to the transcript counts. RaceID was then applied to analyze the transformed data using default parameter values.

In addition, we generated a simulated dataset containing five cell clusters. The two major clusters contained 1000 cells each, whereas the three rare clusters contained 4, 6, and 10 cells, respectively. The gene expression profiles were synthesized by using a strategy similar to that in the RaceID paper [8]. Specifically, gene expression levels within each cell cluster were modeled as negative binomial distributions, with the mean and standard deviation values estimated from an intestinal single-cell RNA-seq dataset through a background noise model [8]. To create different gene expression profiles, for each additional cluster we randomly selected 100 highly expressed genes (mean >10 counts) and 100 not highly expressed genes (mean <10 counts), then we shuffled the gene labels. Principal component analysis confirmed that the five simulated cell clusters were distinguishable, although more than two principal components were required (Additional file 2: Figure S2).

## Abbreviations

CDR, cellular detection rate; DBSCAN, density-based spatial clustering of applications with noise; ESC, embryonic stem cell; GBM, glioblastoma; ISC, intestinal stem cell; LOESS, locally estimated scatterplot smoothing; MASC, mammary gland stem cell; MAST, model-based analysis of single-cell transcriptomics; qPCR, quantitative polymerase chain reaction; RaceID, rare cell type identification; t-SNE, t-distributed stochastic neighbour embedding.

## Additional files

Additional file 1: Table S1. Gini index related statistics for the qPCR dataset from Guo et al. [9]. (XLSX 26 kb) Additional file 2: Table S2. Cluster 5 differentially expressed gene results for qPCR data from Guo et al. study. (XLSX 92 kb) Additional file 3: Table S3. GiniClust clustering membership for Guo et al. study. (XLSX 37 kb) Additional file 4: Figure S1. RaceID cluster result of Guo et al. study qPCR dataset. Each pie chart represents the cell lineage composition of a RaceID cluster. Only the clusters that contain at least one ISC or MASC cell are shown. The total number of cells in each cluster is indicated above each pie chart. (PDF 138 kb) Additional file 5: Figure S2. Comparison of GiniClust and RaceID on the simulated dataset. (A–C) Projection of the simulated data on various principal components; (D) GiniClust identified clusters; (E) decomposition of each simulated rare cluster into GiniClust clusters; (F) RaceID identified clusters; (G) decomposition of each simulated rare cluster into RaceID clusters. (PDF 16879 kb) Additional file 6: Table S4. Gini index related statistics for single-cell RNA-seq data from Klein et al. study. (XLSX 1435 kb) Additional file 7: Table S5. GiniClust clustering membership for Klein et al. study. (XLSX 45 kb) Additional file 8: Table S6. Cluster 2 differentially expressed gene results for single-cell RNA-seq data from Klein et al. study. (XLSX 658 kb) Additional file 9: Figure S3. 2C-like cell marker clustering result of Klein et al. study. (PDF 271 kb) Additional file 10: Table S7. Gini index related statistics for single-cell RNA-seq data from Patel et al. study. (XLSX 1436 kb) Additional file 11: Table S8 GiniClust clustering membership for Patel et al. study. (XLSX 42 kb) Additional file 12: Table S9 Cluster 3 differentially expressed gene results for single-cell RNA-seq data from Patel et al. study. (XLSX 212 kb) Additional file 13: Table S10 Gini index related statistics for single-cell RNA-seq data from Zeisel et al study. (XLSX 999 kb) Additional file 14: Table S11. GiniClust clustering membership for Zeisel et al. study. (XLSX 76 kb) Additional file 15: Figure S4. Expression pattern of hemoglobin genes *Hba-a2_loc1*, *Hba-a2_loc2*, *Hbb-b2*, and *Hbb-bs* in the clusters identified by BackSPIN. The cells that highly express these genes are assigned to different clusters. The plot was generated by using the tool at the Linnarson Lab website: http://linnarssonlab.org/cortex/. (PDF 669 kb) Additional file 16: Table12. Cluster 4 differentially expressed gene results for single-cell RNA-seq data from Zeisel et al. study. (XLSX 233 kb)

## References

[1] Lukk M, Kapushesky M, Nikkila J, Parkinson H, Goncalves A, Huber W (2010). A global map of human gene expression. Nat Biotechnol..

[2] Saadatpour A, Lai S, Guo G, Yuan GC (2015). Single-cell analysis in cancer genomics. Trends Genet..

[3] Stegle O, Teichmann SA, Marioni JC (2015). Computational and analytical challenges in single-cell transcriptomics. Nat Rev Genet..

[4] Zeisel A, Munoz-Manchado AB, Codeluppi S, Lonnerberg P, La Manno G, Jureus A (2015). Brain structure. Cell types in the mouse cortex and hippocampus revealed by single-cell RNA-seq. Science..

[5] Wilson NK, Kent DG, Buettner F, Shehata M, Macaulay IC, Calero-Nieto FJ (2015). Combined single-cell functional and gene expression analysis resolves heterogeneity within stem cell populations. Cell Stem Cell..

[6] Shalek AK, Satija R, Adiconis X, Gertner RS, Gaublomme JT, Raychowdhury R (2013). Single-cell transcriptomics reveals bimodality in expression and splicing in immune cells. Nature..

[7] Brunskill EW, Park JS, Chung E, Chen F, Magella B, Potter SS (2014). Single cell dissection of early kidney development: multilineage priming. Development..

[8] Grun D, Lyubimova A, Kester L, Wiebrands K, Basak O, Sasaki N (2015). Single-cell messenger RNA sequencing reveals rare intestinal cell types. Nature..

[9] Guo G, Luc S, Marco E, Lin TW, Peng C, Kerenyi MA (2013). Mapping cellular hierarchy by single-cell analysis of the cell surface repertoire. Cell Stem Cell..

[10] Saadatpour A, Guo G, Orkin SH, Yuan GC (2014). Characterizing heterogeneity in leukemic cells using single-cell gene expression analysis. Genome Biol..

[11] Orkin SH, Zon LI (2008). Hematopoiesis: an evolving paradigm for stem cell biology. Cell..

[12] Kreso A, Dick JE (2014). Evolution of the cancer stem cell model. Cell Stem Cell..

[13] Plaks V, Koopman CD, Werb Z (2013). Cancer. Circulating tumor cells. Science.

[14] Love MI, Anders S, Kim V, Huber W (2015). RNA-Seq workflow: gene-level exploratory analysis and differential expression. F1000 Res.

[15] Grun D, Kester L, van Oudenaarden A (2014). Validation of noise models for single-cell transcriptomics. Nat Methods..

[16] Gini C, Pizetti E, Salvemini T (1912). Variabilità e mutabilità. Memorie di metodologica statistica.

[17] Ester M, Kriegel HP, Sander J, Xu X. A density-based algorithm for discovering clusters in large spatial databases with noise. In Second Int Conf Knowl Discov Data Min. 1996;226–231.

[18] Maaten L, Hinton G (2008). Visualizing data using t-SNE. J Mach Learn Res..

[19] Klein AM, Mazutis L, Akartuna I, Tallapragada N, Veres A, Li V (2015). Droplet barcoding for single-cell transcriptomics applied to embryonic stem cells. Cell..

[20] Kharchenko PV, Silberstein L, Scadden DT (2014). Bayesian approach to single-cell differential expression analysis. Nat Methods..

[21] Finak G, McDavid A, Yajima M, Deng J, Gersuk V, Shalek AK (2015). MAST: a flexible statistical framework for assessing transcriptional changes and characterizing heterogeneity in single-cell RNA sequencing data. Genome Biol..

[22] Macfarlan TS, Gifford WD, Driscoll S, Lettieri K, Rowe HM, Bonanomi D (2012). Embryonic stem cell potency fluctuates with endogenous retrovirus activity. Nature..

[23] Patel AP, Tirosh I, Trombetta JJ, Shalek AK, Gillespie SM, Wakimoto H (2014). Single-cell RNA-seq highlights intratumoral heterogeneity in primary glioblastoma. Science..

[24] Jain AK, Murty MN, Flynn PJ (1999). Data clustering: a review. ACM Comput Surv..

[25] Biagioli M, Pinto M, Cesselli D, Zaninello M, Lazarevic D, Roncaglia P (2009). Unexpected expression of alpha- and beta-globin in mesencephalic dopaminergic neurons and glial cells. Proc Natl Acad Sci U S A..

[26] Dobin A, Davis CA, Schlesinger F, Drenkow J, Zaleski C, Jha S (2013). STAR: ultrafast universal RNA-seq aligner. Bioinform..

[27] Anders S, Pyl PT, Huber W (2015). HTSeq—a Python framework to work with high-throughput sequencing data. Bioinform..

[28] Ay F, Bailey TL, Noble WS (2014). Statistical confidence estimation for Hi-C data reveals regulatory chromatin contacts. Genome Res..

