# Supplementary figures and images for: GiniClust: detecting rare cell types from single-cell gene expression data with Gini index
Source: Genome Biol. 2016 Jul 1;17:144. doi: 10.1186/s13059-016-1010-4 (PMC4930624; doi:10.1186/s13059-016-1010-4)

Supplemental Figure 1

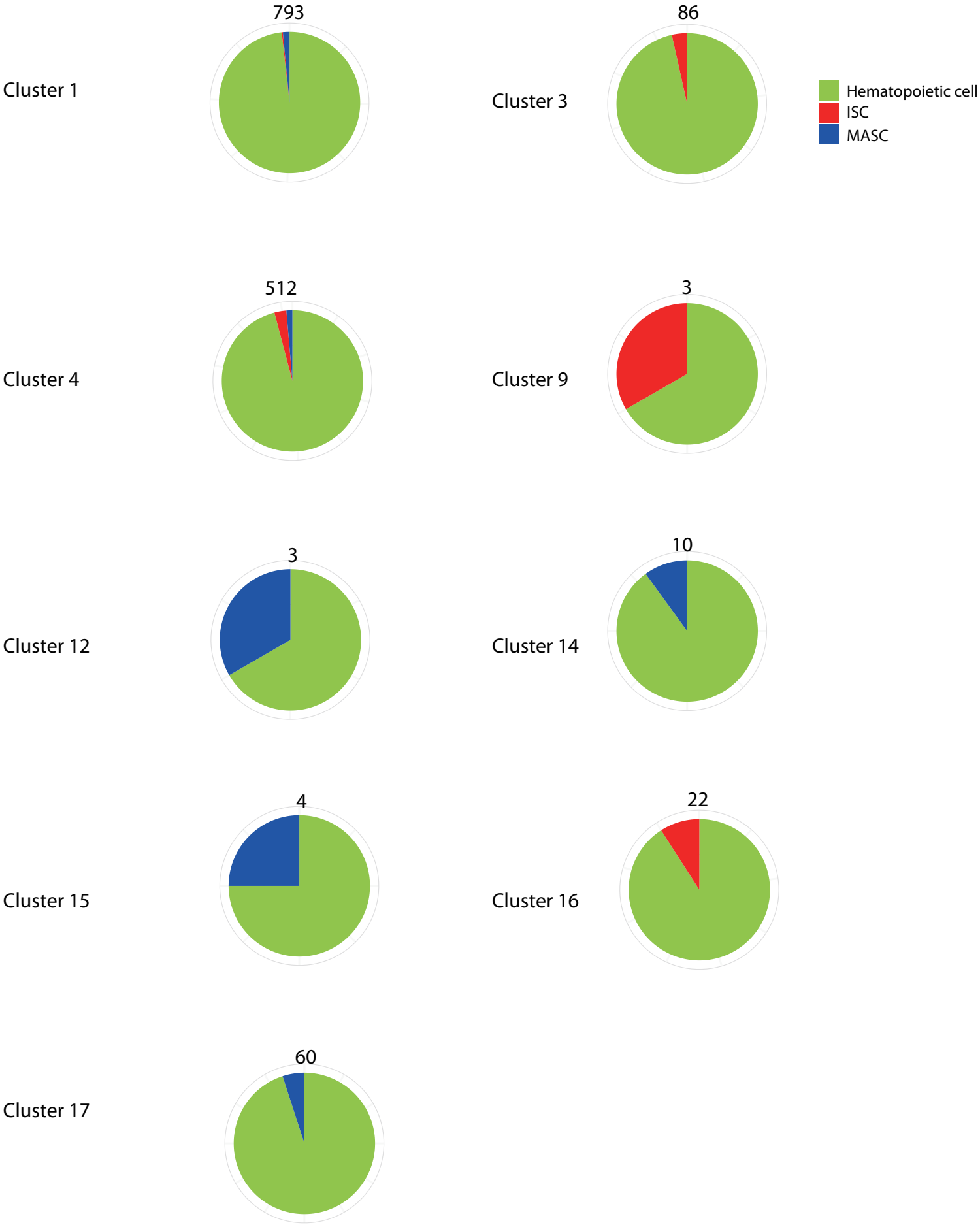

Supplement: Additional file 4: Figure S1. — RaceID cluster result of Guo et al. study qPCR dataset. Each pie chart represents the cell lineage composition of a RaceID cluster. Only the clusters that contain at least one ISC or MASC cell are shown. The total number of cells in each cluster is indicated above each pie chart. (PDF 138 kb) [file 13059_2016_1010_MOESM4_ESM.pdf]

Supplemental Figure 2

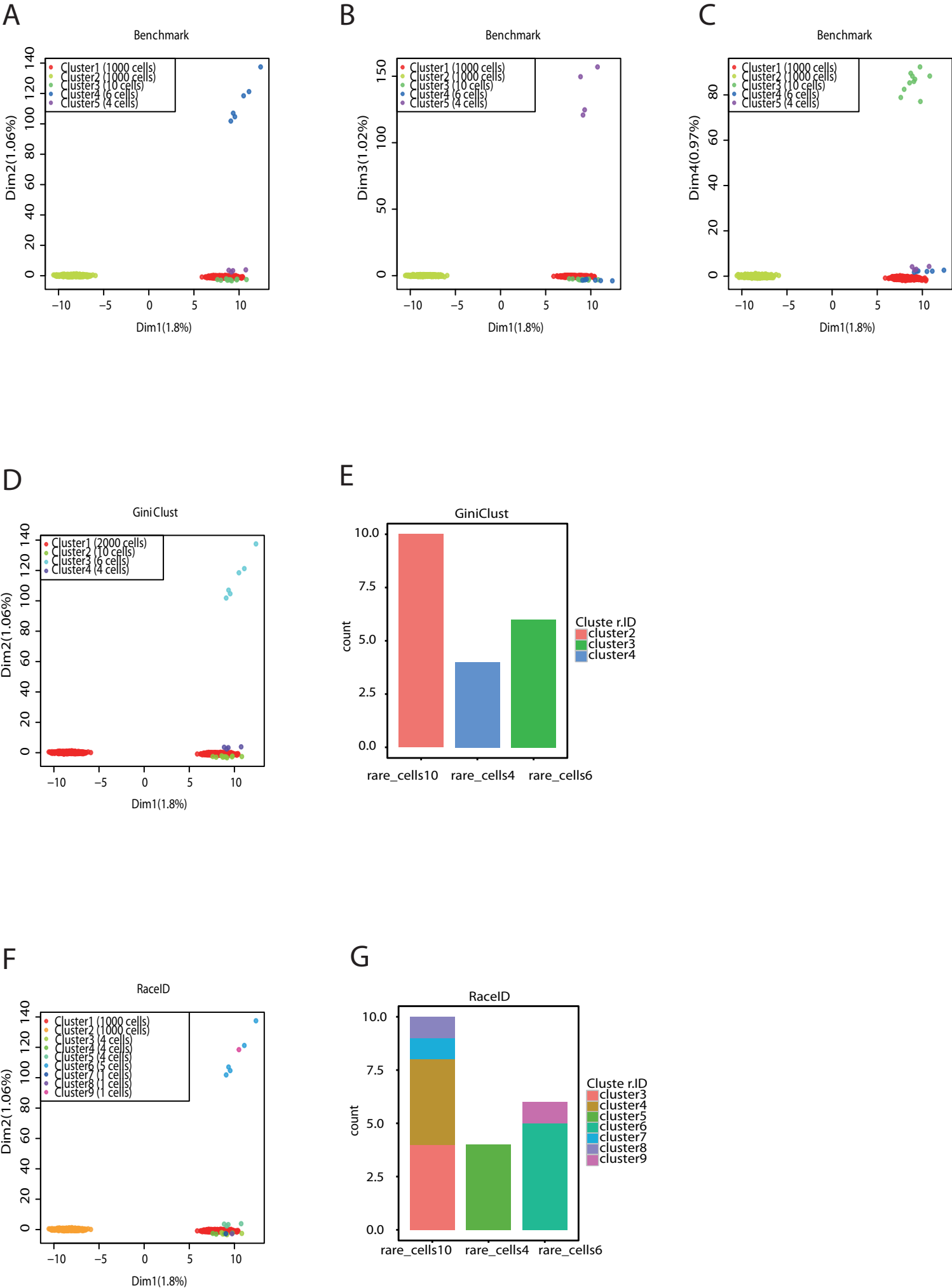

Supplement: Additional file 5: Figure S2. — Comparison of GiniClust and RaceID on the simulated dataset. (A–C) Projection of the simulated data on various principal components; (D) GiniClust identified clusters; (E) decomposition of each simulated rare cluster into GiniClust clusters; (F) RaceID identified clusters; (G) decomposition of each simulated rare cluster into RaceID clusters. (PDF 16879 kb) [file 13059_2016_1010_MOESM5_ESM.pdf]

Supplemental Figure 3

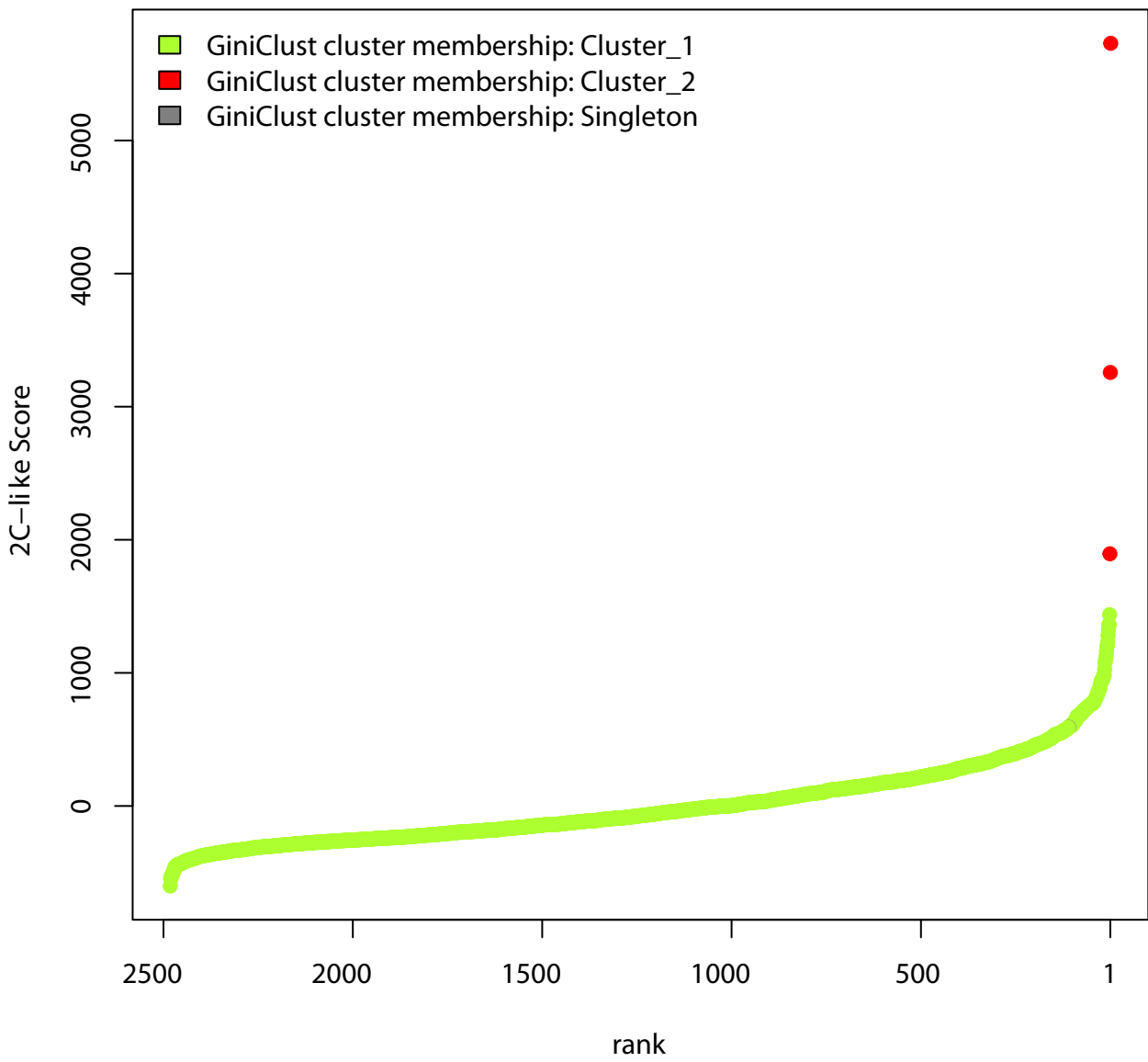

Supplement: Additional file 9: Figure S3. — 2C-like cell marker clustering result of Klein et al. study. (PDF 271 kb) [file 13059_2016_1010_MOESM9_ESM.pdf]

Supplemental Figure 4

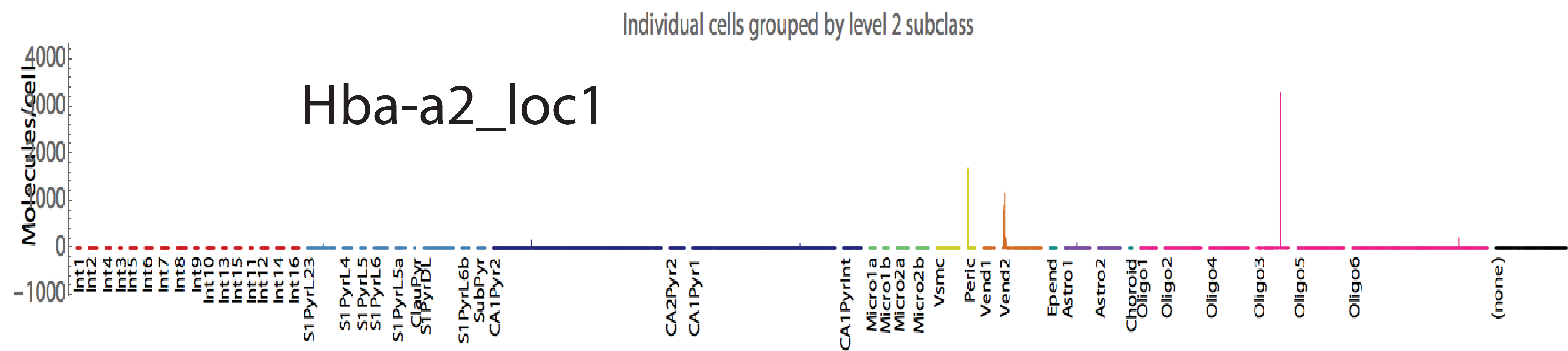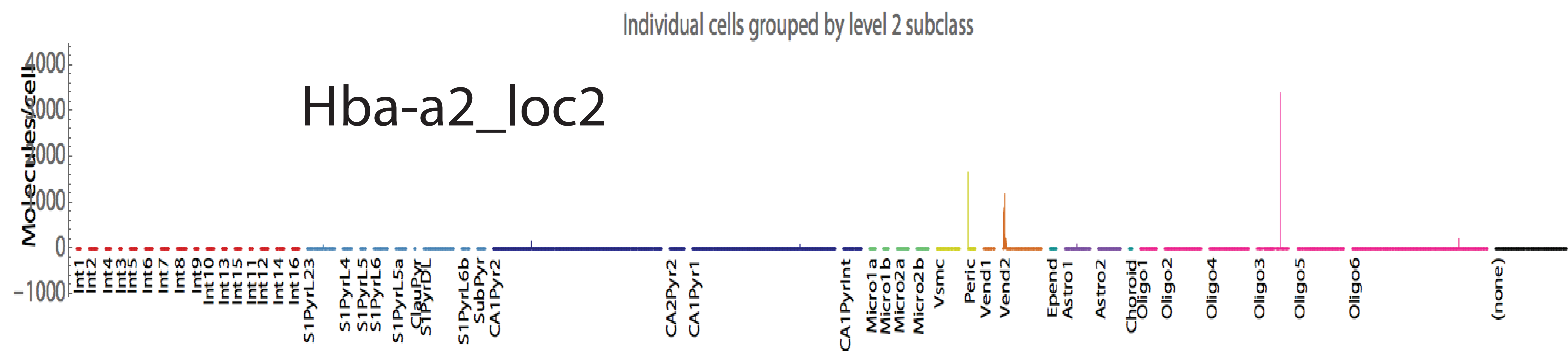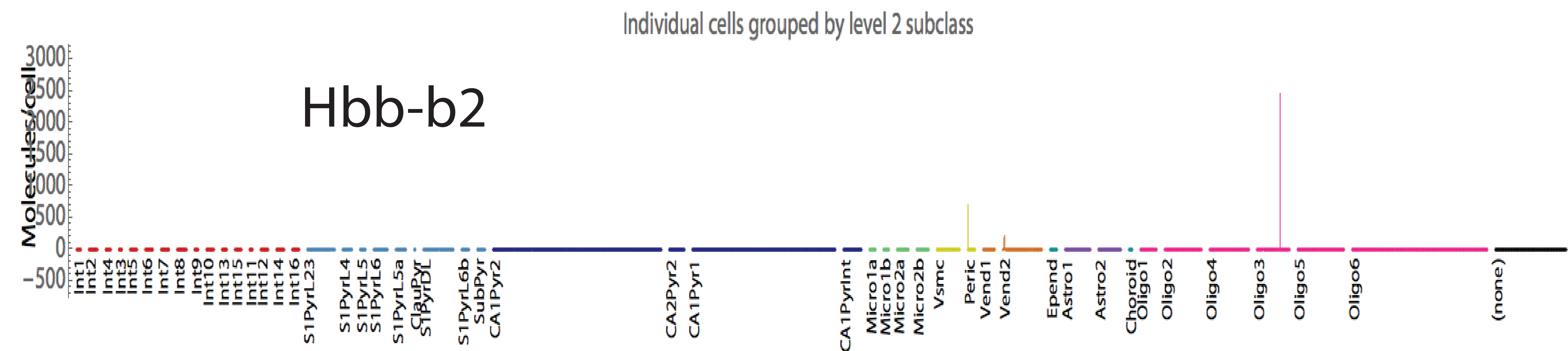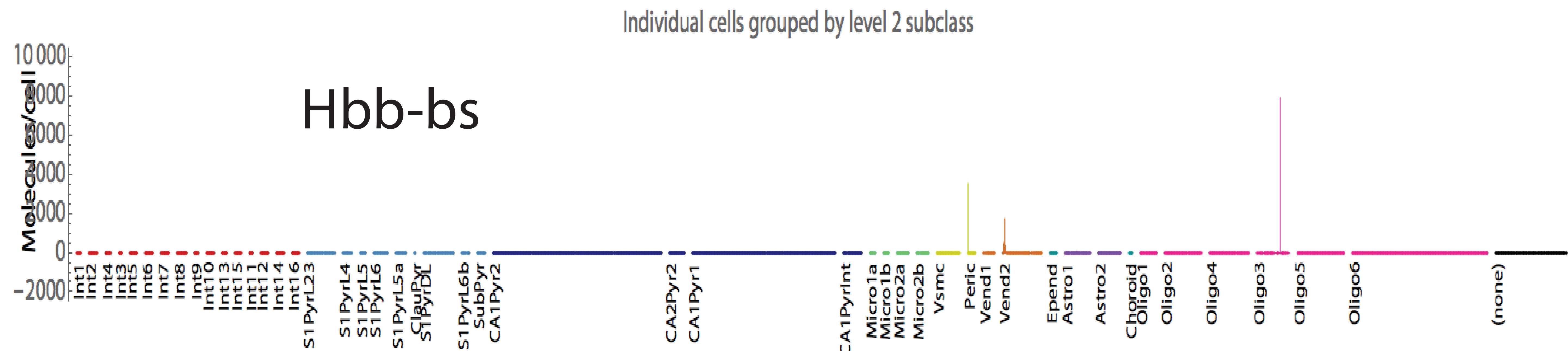

Supplement: Additional file 15: Figure S4. — Expression pattern of hemoglobin genes Hba-a2_loc1, Hba-a2_loc2, Hbb-b2, and Hbb-bs in the clusters identified by BackSPIN. The cells that highly express these genes are assigned to different clusters. The plot was generated by using the tool at the Linnarson Lab website: http://linnarssonlab.org/cortex/. (PDF 669 kb) [file 13059_2016_1010_MOESM15_ESM.pdf]
